# Primary retropharyngeal leiomyosarcoma in a young cat

**DOI:** 10.1177/20551169231164612

**Published:** 2023-04-18

**Authors:** Janusz Jaworski, Aaron Harper, Hayley Crosby-Durrani, Jon Hall

**Affiliations:** 1 Wear Referrals Veterinary Specialists, Bradbury, Stockton-on-Tees, UK; 2 Department of Veterinary Anatomy, Physiology and Pathology, University of Liverpool, Neston, Wirral, UK

**Keywords:** Leiomyosarcoma, computed tomography, retropharyngeal, stridor, surgery

## Abstract

**Case summary:**

An Oriental Shorthair cat, aged 1 year and 6 months, developed progressive stridor and a palpable right ventral cervical mass. Fine-needle aspiration of the mass was inconclusive, while thoracic radiography and CT showed no evidence of metastasis. There was initial improvement in stridor with oral doxycycline and prednisolone treatment, but it recurred 4 weeks later and excisional biopsy was performed. Histopathology with immunohistochemistry diagnosed leiomyosarcoma with incomplete surgical margins. Adjunctive radiation therapy was declined. Repeated physical examination and CT 7 months postoperatively documented no evidence of mass recurrence.

**Relevance and novel information:**

This is the first reported case of retropharyngeal leiomyosarcoma in a young cat with no evidence of local reoccurrence 7 months following an excisional biopsy.

## Introduction

Leiomyosarcomas are uncommon, malignant tumours arising from smooth muscle cells^1^ and have been reported in the duodenum,^2^ iliocaecocolon,^3^ large intestine,^1^ urinary bladder,^4^ stomach,^5^ oesophagus,^6^ spleen,^1^ uterus,^7^ pancreas,^8^ liver,^9^ kidney,^10^ vulva,^11^ eye,^12^ cutaneous smooth muscle,^13,14^ and dermal interphalangeal region^15^ and heart.^16^ They are frequently non-encapsulated and invasive tumours.^1^ Histological features vary from densely packed, relatively homogeneous spindle cells with the appearance of smooth muscle to more pleomorphic ovoid or round cells.^1^

Surgery is the recommended treatment of choice and the prognosis depends on the anatomical location of the tumour and the presence of metastasis.^2–16^

To the authors’ knowledge, this report describes a novel diagnosis of retropharyngeal leiomyosarcoma in a young cat with a good outcome following marginal surgical excision. This tumour should be considered as a differential diagnosis for a retropharyngeal mass in the cat.

## Case description

**Figure 1:**
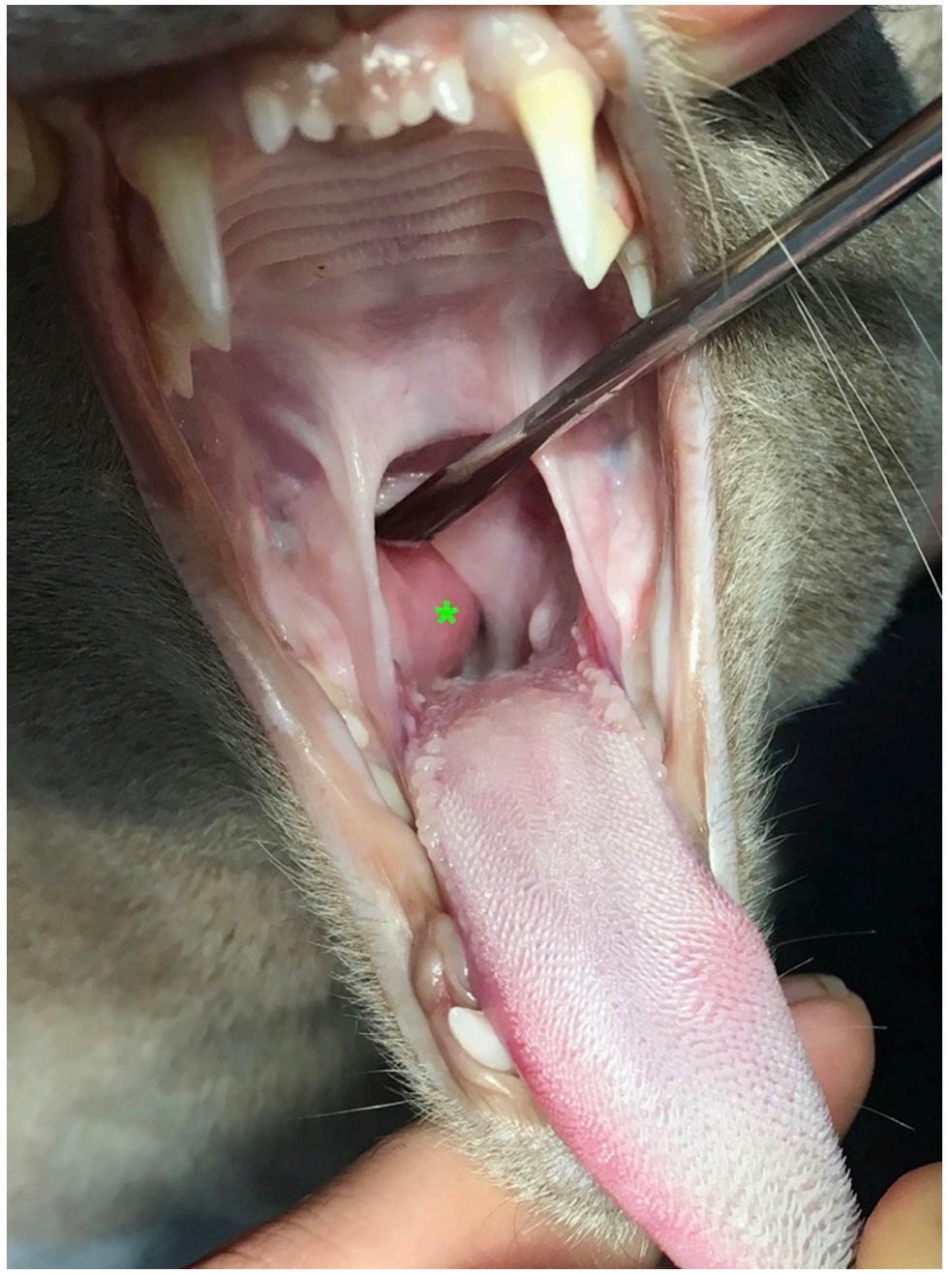
Intraoral image with dorsal elevation of the soft palate demonstrating a submucosal retropharyngeal mass (green star)

**Figure 2:**
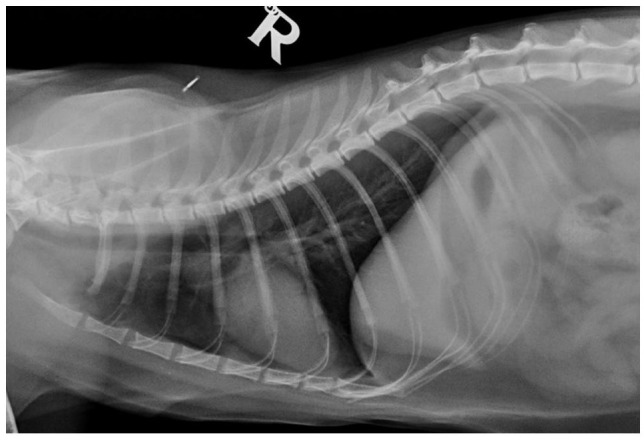
Right lateral thoracic radiograph demonstrating a caudodorsal interstitial lung pattern

A neutered male Oriental Shorthair cat, aged 1 year and 6 months, was seen by the primary care practice (PCP) with stridor that had progressively worsened over the course of 3 weeks. Clinical examination was unremarkable. Haematology and serum biochemistry were within normal limits. The patient was sedated and examination of the oral cavity revealed a large retropharyngeal mass (Figure 1). A fine-needle aspirate of the mass was obtained and thoracic radiographs showed a caudodorsal bronchial lung pattern (Figure 2), prompting bronchoalveolar lavage (BAL) to be performed under general anaesthesia.

The BAL results were consistent with mixed eosinophilic inflammation with suspected allergic disease. Fine-needle aspiration (FNA) results were inconclusive. The cat received a subcutaneous injection of dexamethasone (0.2 mg/kg [Colvasone; Norbrook]).

**Figure 3:**
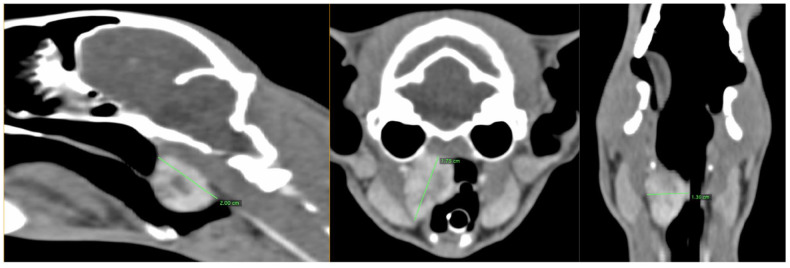
Contrast-enhanced CT multiplanar reconstruction using soft tissue windowing, demonstrating right-sided retropharyngeal mass (green marker)

CT of the head and thorax were performed by the PCP and interpreted by a diagnostic imaging specialist. CT revealed a discrete right retropharyngeal/tonsillar mass with no local infiltration into the surrounding tissues, ipsilateral medial retropharyngeal lymphadenopathy and unspecific pulmonary changes (Figure 3).

The cat was referred to Wear Referrals, Stockton-on-Tees, UK, and an approximately 1 × 2 cm firm soft tissue mass was palpated on the right lateral neck immediately ventral to the right tympanic bulla and cranioventral to the wing of the atlas, displacing the larynx to the left. The owner reported that the clinical signs were stable. The cat had moderate stridor but no other abnormalities on clinical examination. Owing to the cat’s young age, lack of conclusive evidence of neoplasia and the possibility of an inflammatory diagnosis, the cat was prescribed doxycycline (10 mg/kg PO q24h [Ronaxan; Merial]) and prednisolone (1 mg/kg PO q24h [Prednicare; Animalcare]) for 4 weeks. The cat was re-examined 4 weeks later and the stridor had improved, although the mass was unchanged in size. Medications were continued for a further 5 weeks. On re-examination, the stridor had recurred and excisional biopsy was recommended.

The cat was premedicated with medetomidine (5 μg/kg IV [Dorbene; Zoetis]) and butorphanol (0.3 mg/kg IV [Torbugesic; Zoetis]). Cefuroxime (20 mg/kg IV [Zinacef; GSK]) was administered 30 mins prior to surgery. General anaesthesia was induced using alfaxalone (4 mg/kg IV [Alfaxan; Jurox]). Intubation was achieved using a 3.5 mm endotracheal tube, and anaesthesia was maintained with isoflurane (IsoFlo; Zoetis) in 100% oxygen. Analgesia was provided with fentanyl (0.2 μg/kg/min IV [Fentadon; Dechra]) as a constant rate infusion.

The patient was positioned in dorsal recumbency, with the neck extended and supported by a sandbag. A ventral midline cervical incision was created, and a combination of blunt and sharp dissection through subcutaneous tissue and sphincter coli muscle was performed to identify the mass ventral to the right tympanic bulla. The mass was marginally dissected from the local neurovascular structures (branches from the lingual vein, the hypoglossal nerve), muscles (digastricus, styloglossus and hypoglossus) and other adjacent anatomy (the lateral surface of the larynx and ventral surface of the right tympanic bulla) using a combination of delicate, blunt and sharp dissection and bipolar electrosurgery. Lavage and routine closure were performed. The mass was submitted for histopathology.

Intravenous fentanyl was discontinued immediately postoperatively, and the cat received buprenorphine (0.02 mg/kg IV q6h [Buprecare; Animalcare]). The cat was discharged 18 h postoperatively with prednisolone (0.6 mg/kg PO q24h [Prednicare; Animalcare]) for 4 weeks with a tapering dose and buprenorphine (0.02 mg/kg sublingually q8h [Buprecare; Animalcare]) for 3 days. The patient was reviewed 4 weeks following the surgery. The owner reported that the stridor had fully resolved.

**Figure 4:**
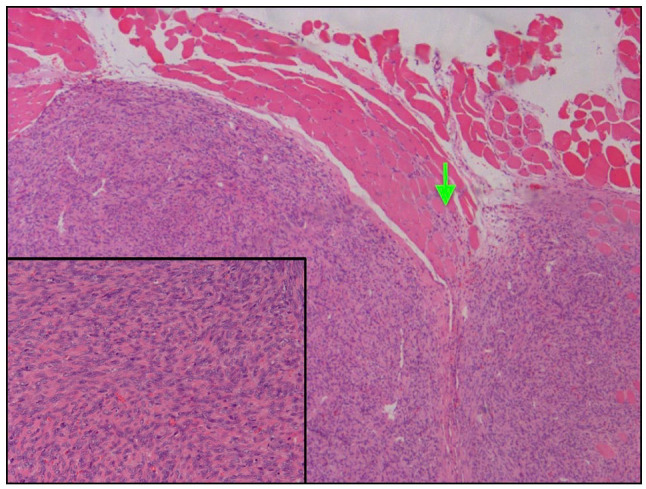
Low-power microscopic view (× 40) of leiomyosarcoma infiltrating adjacent skeletal muscle (green arrow; haematoxylin and eosin stain). The inset represents a high-powered view (× 200) of mesenchymal neoplastic cells

**Figure 5:**
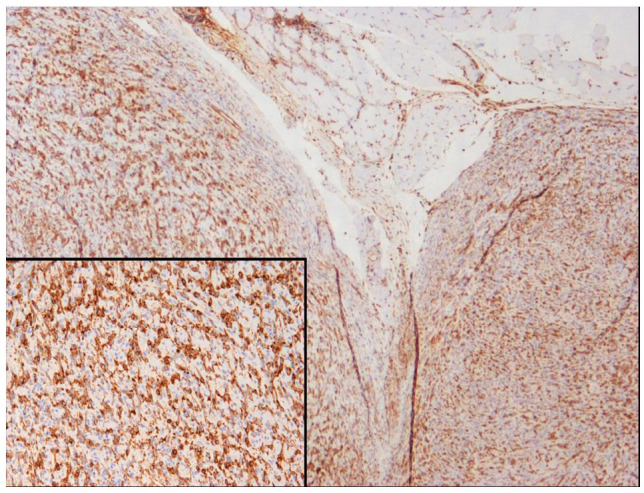
Low-power microscopic view (× 40) of the vimentin immunohistochemistry of leiomyosarcoma. The inset is a high-power view of the neoplastic cells (× 200). The brown colour indicates positive labelling of the neoplastic cells

**Figure 6:**
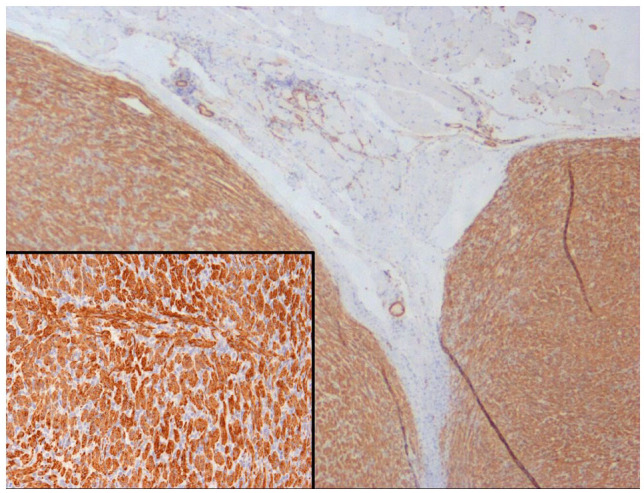
Low-power microscopic view (× 40) of the alpha smooth muscle actin immunohistochemistry. The inset is a high-power view of the neoplastic cells (× 200). The brown colour indicates strong and specific positive labelling of the neoplastic cells

**Figure 7:**
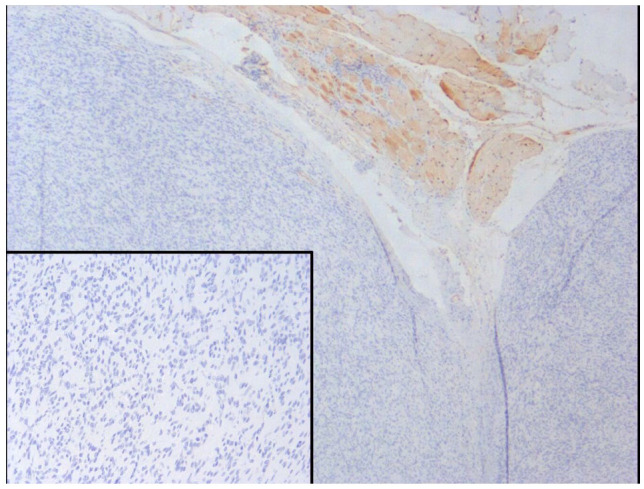
Low-power microscopic view (× 40) of the myoglobin immunohistochemistry. The inset is a high-power view of neoplastic cells (× 200). The neoplastic cells show negative immunohistochemistry labelling for myoglobin

Histology described an infiltrative but partially encapsulated malignant tumour of mesenchymal origin. Neoplastic cells were spindloid with indistinct cell borders, abundant eosinophilic cytoplasm and elongated ovoid nuclei, arranged in bundles and whorls within fibrovascular stroma. Chromatin was finely stippled to coarsely clumped with 1–3 small nucleoli per cell. Neoplastic cells exhibited moderate to marked anisocytosis and anisokaryosis with a mitotic count of 1 per 10 high-power fields at × 200 magnification and an area of 2.37 mm^2^ (Figure 4). Immunohistochemistry revealed diffuse strong staining of the neoplastic cells with antivimentin (1:500 dilution [Monoclonal Mouse Anti-Human Vimentin Clone; Dako]), antidesmin (1:100 dilution [Monoclonal Mouse Anti-Human Desmin Clone; Dako]) and anti-alpha smooth muscle actin (1:100 dilution [Monoclonal Mouse Anti-Human Smooth Muscle Actin Clone; Dako]) antibodies at × 200 magnification (Figures 5 and 6), consistent with a leiomyosarcoma. The neoplastic cells showed negative immunohistochemistry labelling for myoglobin (1:100 dilution [Monoclonal Mouse Anti-Human Myoglobin Clone; Dako]) (Figure 7).

While gross cytoreduction had been achieved to maximise diagnostic value and therapeutic benefit for the cat, histologically clear margins were not complete. Adjunctive radiation therapy of the primary tumour location was discussed, but the owners declined this option.

**Figure 8:**
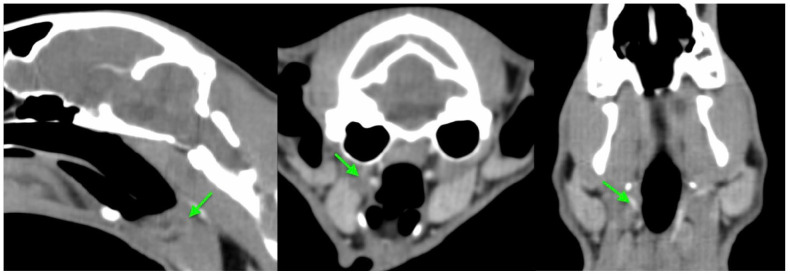
Contrast-enhanced CT multiplanar reconstruction using soft tissue windowing demonstrating lack of tumour reoccurrence 7 months following the surgery. Green arrows show previous location of the mass

The cat was re-examined 7 months following surgery. The owner reported no recurrence of stridor and the cat was normal on examination. Repeat contrast-enhanced CT 16-slice helical scans (Somaton Emotion; Siemens) of the head and thorax was performed. CT revealed no evidence of primary mass recurrence (Figure 8), static right retropharyngeal mild lymphadenopathy and a poorly defined, mildly contrast-enhancing soft tissue opacity within the cranial mediastinum (possible mildly enlarged mediastinal lymph node). Further investigation was declined.

## Discussion

This report describes the first case of retropharyngeal leiomyosarcoma in a young cat and should be considered as a differential diagnosis for a mass in this location.

The main presenting clinical sign was stridor, likely due to the partial obstruction of the upper respiratory tract and displacement of the larynx, and the mass could be readily identified by palpation and direct visualisation.

As far as we are aware, the youngest cats diagnosed with leiomyosarcoma were one cat aged 3 years and 9 months, which was diagnosed with oesophageal angioleiomyosarcoma,^6^ followed by a 4-year-old cat with documented primary bladder leiomyosarcoma.^4^ While most of the reported cases feature middle-aged to older cats,^1,3,11,15^ the cat in this report was 1 year and 6 months old, which represents an uncommon, early presentation for this type of tumour.

The initial lack of a definitive diagnosis by FNA, the young age of the cat and the findings of eosinophilic pulmonary disease influenced the initial decision to provide a therapeutic trial with oral antibiotics and steroids. The initial improvement in clinical signs may be due to reduced peritumoral inflammation. The failure of the mass to respond to treatment then prompted the decision for a more invasive procedure. Excisional biopsy was elected over Tru-cut or incisional biopsy as the clinical signs of stridor were likely due to the mass effect on the trachea and therefore a planned marginal excision would provide therapeutic benefit.

Immunohistochemistry was necessary because the histopathological features of the mass were similar to rhabdomyosarcoma and fibrosarcoma. The location of the mass, particularly in a young cat, also meant that other differential diagnoses would be more likely than leiomyosarcoma. Leiomyosarcomas that develop in the haired skin and subcutaneous tissues are thought to arise from smooth muscle associated with the vasculature or arrector pili muscles.^1^ The oesophagus of the cat appeared radiologically normal and would only be expected to contain smooth muscle in the intrathoracic portion. It is possible that this tumour may have arisen from vascular smooth muscle in this area. An additional differential diagnosis considered in this case was laryngeal tumour^17^ or cyst.^18^ Oral examination and CT ruled out a laryngeal origin of this mass.

Postoperatively, the cat was prescribed prednisolone to decrease postoperative inflammation and respiratory tract obstruction following the extensive dissection in the area, and because eosinophilic lung disease had previously been noted on BAL (with an initial improvement in clinical signs when treated with prednisolone and oral antibiotics). It is unclear whether the treatment with prednisolone had any effect on the outcome, although corticosteroids are not generally considered to have antineoplastic action on mesenchymal tumours. Adjunctive radiation therapy has not been extensively reported for incompletely excised feline leiomyosarcomas, although it would be considered a beneficial therapy for residual microscopic disease. However, radiotherapy was declined by the owners.

Long-term follow-up data for cats treated for leiomyosarcoma are largely lacking. The reported prognosis following the surgical removal of the tumour varies from 1 month^7^ to 48 months.^4^ Numerous case reports documented the short-term prognosis and no tumour reoccurrence 5,^8^ 6^6,12,15^ and 10 months^5^ postoperatively, respectively. At the time of writing, the cat had shown no signs of tumour recurrence; the presence of mild unilateral retropharyngeal lymphadenopathy and cranial mediastinal lymphadenopathy could indicate metastatic disease, although leiomyosarcoma do not typically metastasise via the lymphatic route. Other causes of the imaging findings (eg, reactive hyperplasia or normal anatomical variance) were considered more likely. Continued surveillance of the cat and, ultimately, post-mortem examination are required for complete information.

## Conclusions

Leiomyosarcoma should be a differential diagnosis for cats presenting with a retropharyngeal mass. A good medium-term outcome can be achieved with marginal excision.
